# Supplementary material for: Complement activation assessed by C3bc and C5b-9 terminal complex as diagnostic biomarkers for deep vein thrombosis
Source: PLoS One. 2025 Oct 6;20(10):e0333206. doi: 10.1371/journal.pone.0333206 (PMC12500123; doi:10.1371/journal.pone.0333206)
Supplement: S1 Table — (DOCX) [file pone.0333206.s001.docx]

**S1 Table** Definitions of extracted variables and diagnostic accuracy estimates.

| **Variable** | **Definitions used for data extraction and data analyses** |
| --- | --- |
| Sex | Categorical variable, male = 1 or female = 0. Biological gender reported by Akershus University Hospital electronic medical record system. |
| Symptom duration | Continuous variable, minimum value 1 hour and maximum value 99 hours. Self-reported onset of DVT-related symptoms (lower limb redness, swelling, venous ectasia and/or pain). If days from symptom onset were reported, such as one, two, three etc. days ago, the variable were coded as 24, 48, 72 etc. hours. |
| Surgery/trauma | Categorical variable, yes = 1 or no = 0. “Yes” if self-reported by the patient or stated in the medical record medical of recent trauma or surgery within the last 3 months prior to study inclusion. |
| Family history of VTE | Categorical variable, yes = 1 or no = 0. “Yes” if self-reported by the patient a family history of VTE, *i.e.* at least one first-degree relative in whom had an VTE before turning 60 years old. |
| Hormonal therapy | Categorical variable, yes = 1 or no = 0. “Yes” if self-reported by the patient or stated in the medical record medical, usage of estrogen-containing oral contraceptives, hormonal replacement therapy with estrogens or selective estrogen receptor modulators (SERMs). |
| Smoker | Categorical variable, yes = 1 or no = 0. “Yes” if self-reported by the patient or stated in the medical record medical of current tobacco smoking. |
| BMI ≥ 25 kg/m^2^ | Categorical variable, yes = 1 or no = 0. “Yes” if emergency department physician reported that the patient was "overweight”, “obese” or reporting av BMI ≥ 25 kg/m^2^ in the medical record. Also “yes” if weight and height were reported in the medical record system at admission or within 3 months before admission. BMI was then calculated (by author VEW). Approximately half the “yes” values were based on physicians reporting that the patient was “overweight”/”obese” in the medical record, and the other half from BMI calculations based on height and weight. |
| Pregnant or postpartum | Categorical variable, yes = 1 or no = 0. “Yes” if self-reported by the patient or stated in the medical record medical, of being pregnant or ≤ 8 weeks since the patient had given birth at the timepoint of inclusion. |
| Known thrombophilia | Categorical variable, yes = 1 or no = 0. “Yes” if self-reported by the patient or stated in the medical record medical of an inherited or acquired thrombophilia disorder. |
| Cancer  1.Previous  2.Active | Categorical variable, yes = 1 or no = 0.   1. “Yes” if the patient was not receiving ongoing cancer treatment (surgery, immunochemotherapy or radiation) at timepoint of inclusion or if the cancer diagnosis was determined ≥ 2 years before the study inclusion date. 2. “Yes” if the patient was still receiving ongoing cancer treatment (surgery, immunochemotherapy or radiation) at timepoint of inclusion or if the cancer diagnosis was determined < 2 years before the study inclusion date. |
| Myocardial infarction | Categorical variable, yes = 1 or no = 0. “Yes” if the patients reported themselves or evidence in their medical record of any type of previous myocardial infarction. |
| COPD | Categorical variable, yes = 1 or no = 0. “Yes” if self-reported by the patient or stated in the medical record of chronic obstructive lung disease. |
| Heart failure | Categorical variable, yes = 1 or no = 0. “Yes” if self-reported by the patient or stated in the medical record of any type of heart failure. |
| Stroke | Categorical variable, yes = 1 or no = 0. “Yes” if self-reported by the patient or stated in the medical record of any type of previous stroke. |
| Connective tissue disorder | Categorical variable, yes = 1 or no = 0. “Yes” if self-reported by the patient or stated in the medical record of any type of systemic (inherited or inflammatory) connective tissue disorder (e.g. Ehler Danlos, systemic lupus erythematosus, Marfans syndrome, systemic scleroderma etc. ) |
| Antithrombotic therapy  1.DOAC  2.LMWH  3.ADP receptor antagonist  4.Acetylsalisyl acid | Categorical variable, yes = 1 or no = 0.  1. and 2. “Yes” to usage of anticoagulants (direct oral anticoagulants, low-molecular weight heparin or warfarin) if a single dose were prescribed to the patient by the primary health care or emergency department physician while awaiting diagnostic tests for venous thromboembolism to be performed.  3. and 4. “Yes” if self-reported by the patient or stated in the medical record of ongoing treatment with an ADP receptor antagonist or acetylsalicylic acid. |
| Isolated DVT | Categorical variable, yes = 1 or no = 0. “Yes” if the patient was diagnosed with deep vein thrombosis without radiological evidence of concurrent pulmonary embolism. |
| DVT and PE | Categorical variable, yes = 1 or no = 0. “Yes” if the patient was diagnosed with deep vein thrombosis with radiological evidence of concurrent pulmonary embolism. |
| Proximal DVT | Categorical variable, yes = 1 or no = 0. “Yes” if the compression ultrasound description describes a thrombus involving the vena poplitea and/or more proximal veins. Vena cava inferior/superior are not anatomically available for visualization and compression with this radiological technique. |
| Distal DVT | Categorical variable, yes = 1 or no = 0. “Yes” if the compression ultrasound description describes a thrombus under level of the vena poplitea. |
| Missed VTE | Categorical variable, yes = 1 or no = 0. “Yes” stated in the medical record a VTE diagnosis within 3 months after study inclusion date. |
| Cut-off value | The value of the index in which the measurement is higher or lower than this value; the result will either be positive or negative test result. |
| Sensitivity | - Probability that a diseased individual will receive a positive test result.  - a / (a+c) = true positives / (true positives + false negatives) |
| Specificity | - Probability that a non-diseased individual will receive a negative test result.  - d / (b+d) = true negatives / (true negatives + false positive) |
| Positive predictive value | - Probability that a individual with a positive test result originates from a diseased person.  - a / (a+b) = true positives / (true positives + false positives) |
| Negative predictive value | - Probability that an individual with a negative test result originates from a non-diseased person.  - d / (c+d) = true negatives / (true negatives + false negatives) |

Abbreviations: DVT: deep vein thrombosis, VTE: venous thromboembolism, BMI: body mass index, COPD: chronic obstructive pulmonary disease, DOAC: direct oral anticoagulants, LMWH: low molecular weight heparin, ADP: adenosine-diphosphate, PE: pulmonary embolism.
