# Supplementary material for: Complement activation assessed by C3bc and C5b-9 terminal complex as diagnostic biomarkers for deep vein thrombosis
Source: PLoS One. 2025 Oct 6;20(10):e0333206. doi: 10.1371/journal.pone.0333206 (PMC12500123; doi:10.1371/journal.pone.0333206)
Supplement: S2 Table — List and frequency of all international classification of diseases version 10 (ICD-10) registered in Akershus university hospital medical record system of patients in whom deep vein thrombosis were excluded. (DOCX) [file pone.0333206.s002.docx]

**S2 Table. Registered diagnoses of non-deep vein thrombosis patients.** List and frequency of all international classification of diseases version 10 (ICD-10) registered in Akershus university hospital medical record system of patients in whom deep vein thrombosis was excluded.

| **ICD-10 diagnoses of non-DVT-patients (n=263)** | | |
| --- | --- | --- |
| M76.6 | Achilles tendinitis | 3 |
| I21.4 | Acute subendocardial myocardial infarction^1^ | 1 |
| L50.0 | Allergic urticaria | 1 |
| I72.4 | Aneurysm and dissection of artery of lower extremity | 1 |
| M13.96 | Arthritis, unspecified;calf/knee | 1 |
| D17.2 | Benign lipomatous neoplasm of skin and subcutaneous tissue of limbs | 1 |
| M77.3 | Calcaneal spur | 2 |
| L03.9 | Cellulitis, unspecified | 3 |
| S80.0 | Contusion of knee | 1 |
| S80.1 | Contusion of other and unspecified parts of lower leg | 1 |
| R06.0 | Dyspnoea^1^ | 1 |
| A46 | Erysipelas | 2 |
| M10.0 | Idiopathic gout | 5 |
| M10.07 | Idiopathic gout;ankle/foot/toe | 1 |
| M12.46 | Intermittent hydrarthrosis;calf/knee | 1 |
| C77.2 | Intra-abdominal lymph nodes | 1 |
| R60.0 | Localized oedema | 5 |
| R22.4 | Localized swelling, mass and lump, lower limb | 4 |
| R22.9 | Localized swelling, mass and lump, unspecified | 1 |
| A69.2 | Lyme disease | 1 |
| I89.0 | Lymphoedema, not elsewhere classified | 1 |
| M62.6 | Muscle strain | 1 |
| M79.16 | Myalgia;calf/knee | 2 |
| R60.9 | Oedema, unspecified | 1 |
| M23.2 | Other meniscus derangements | 1 |
| M12.8 | Other specific arthropathies, not elsewhere classified | 1 |
| M25.5 | Pain in joint | 3 |
| M25.56 | Pain in joint;calf/knee | 2 |
| M79.67 | Pain in limb | 9 |
| M25.57 | Pain in limb;ankle/foot/toe | 2 |
| M79.66 | Pain in limb;calf/knee | 130 |
| M79.65 | Pain in limb;pelvis/thigh | 1 |
| M76.7 | Peroneal tendinitis | 1 |
| I80.2 | Phlebitis and thrombophlebitis of other deep vessels of lower extremities | 9 |
| I80.8 | Phlebitis and thrombophlebitis of other sites | 6 |
| I80.0 | Phlebitis and thrombophlebitis of superficial vessels of lower extremities | 6 |
| I80.9 | Phlebitis and thrombophlebitis of unspecified site | 1 |
| M79.55 | Residual foreign body in soft tissue;pelvis/thigh | 1 |
| M66.0 | Rupture of popliteal cyst | 34 |
| C79.5 | Secondary malignant neoplasm of bone and bone marrow | 1 |
| M79.9 | Soft tissue disorder, unspecified | 1 |
| R23.3 | Spontaneous ecchymoses | 1 |
| M71.2 | Synovial cyst of popliteal space [Baker] | 4 |
| I83.9 | Varicose veins of lower extremities without ulcer or inflammation | 6 |
| I87.2 | Venous insufficiency (chronic)(peripheral) | 1 |
| ^1^ = patient had symptoms of both DVT and PE. | | |

Abbreviations: DVT: deep vein thrombosis, ICD-10: international classification of diseases version 10, PE: pulmonary embolism.
