# Supplementary material for: Complement activation assessed by C3bc and C5b-9 terminal complex as diagnostic biomarkers for deep vein thrombosis
Source: PLoS One. 2025 Oct 6;20(10):e0333206. doi: 10.1371/journal.pone.0333206 (PMC12500123; doi:10.1371/journal.pone.0333206)
Supplement: S3 Table — Contingency table displaying diagnostic accuracy estimates of D-dimer using the cut-off value of ≥ 0.50 mg/L. (DOCX) [file pone.0333206.s003.docx]

**S3 Table.** **Contingency table of D-dimer**. Contingency table displaying diagnostic accuracy estimates of D-dimer using the cut-off value of ≥ 0.50 mg/L.

| D-dimer | | | | | | |
| --- | --- | --- | --- | --- | --- | --- |
| D-dimer AUC = 0.92 (95% confidence interval: 0.89 – 0.95) | | Reference standard CUS | | | |  |
|  |  | Positive | | Negative | | Sum: |
| D-dimer, cut-off:  ≥ 0.50 mg/L | Positive | 100 | | 150 | | 250 |
|  | Negative | 3 | | 109 | | 112 |
|  | Sum: | 103 | | 259 | | 362 |
| Sensitivity: 97% | Specificity: 42% | | PPV: 40% | | NPV: 97% | |

Abbreviations: AUC: area under the curve, CUS: Compression ultrasound, PPV: positive predictive value, NPV: negative predictive value.
