# Supplementary material for: Complement activation assessed by C3bc and C5b-9 terminal complex as diagnostic biomarkers for deep vein thrombosis
Source: PLoS One. 2025 Oct 6;20(10):e0333206. doi: 10.1371/journal.pone.0333206 (PMC12500123; doi:10.1371/journal.pone.0333206)
Supplement: Attachment — Submitted filename: 20250714 Reviewresponse rebuttal letter PLOS ONE.docx [file pone.0333206.s005.docx]

Responses to comments from the editor:

**1.** **Please ensure that your manuscript meets PLOS ONE's style requirements, including those for file naming. The PLOS ONE style templates can be found at https://journals.plos.org/plosone/s/file?id=wjVg/PLOSOne_formatting_sample_main_body.pdf and https://journals.plos.org/plosone/s/file?id=ba62/PLOSOne_formatting_sample_title_authors_affiliations.pdf**

Response: We have edited manuscript, supporting information and figure-file names according to PLOS ONE’s style requirements.

**2. In the online submission form, you indicated that data cannot be shared publicly according to Norwegian law regulations on research using human subjects where health information has been extracted. Relevant requests for access data need to approved by the Regional Committee for Medical Research Ethics North Norway and Patient Data Protection Office at Akerhus University Hospital in Norway.**

**All PLOS journals now require all data underlying the findings described in their manuscript to be freely available to other researchers, either 1. In a public repository, 2. Within the manuscript itself, or 3. Uploaded as supplementary information.
This policy applies to all data except where public deposition would breach compliance with the protocol approved by your research ethics board. If your data cannot be made publicly available for ethical or legal reasons (e.g., public availability would compromise patient privacy), please explain your reasons on resubmission and your exemption request will be escalated for approval.**

**3. We note that you have indicated that there are restrictions to data sharing for this study. For studies involving human research participant data or other sensitive data, we encourage authors to share de-identified or anonymized data. However, when data cannot be publicly shared for ethical reasons, we allow authors to make their data sets available upon request. For information on unacceptable data access restrictions, please see http://journals.plos.org/plosone/s/data-availability#loc-unacceptable-data-access-restrictions.**

**Before we proceed with your manuscript, please address the following prompts:**

**a) If there are ethical or legal restrictions on sharing a de-identified data set, please explain them in detail (e.g., data contain potentially identifying or sensitive patient information, data are owned by a third-party organization, etc.) and who has imposed them (e.g., a Research Ethics Committee or Institutional Review Board, etc.). Please also provide contact information for a data access committee, ethics committee, or other institutional body to which data requests may be sent.**

**b) If there are no restrictions, please upload the minimal anonymized data set necessary to replicate your study findings to a stable, public repository and provide us with the relevant URLs, DOIs, or accession numbers. Please see http://www.bmj.com/content/340/bmj.c181.long for guidelines on how to de-identify and prepare clinical data for publication. For a list of recommended repositories, please see https://journals.plos.org/plosone/s/recommended-repositories. You also have the option of uploading the data as Supporting Information files, but we would recommend depositing data directly to a data repository if possible.**

**Please update your Data Availability statement in the submission form accordingly.**

**4. Please remove all personal information, ensure that the data shared are in accordance with participant consent, and re-upload a fully anonymized data set.**

**Note: spreadsheet columns with personal information must be removed and not hidden as all hidden columns will appear in the published file.**

**Additional guidance on preparing raw data for publication can be found in our Data Policy (https://journals.plos.org/plosone/s/data-availability#loc-human-research-participant-data-and-other-sensitive-data) and in the following article: http://www.bmj.com/content/340/bmj.c181.long.**

Response to comment 2, 3 and 4: Thank you for this comment. We acknowledge PLOS ONE’s effort to make medical research more transparent through requesting all underlying data to be made available when it is ethically and legally possible. Unfortunately, as described upon submission, we cannot make out data publicly available for legal reasons. Norwegian laws regarding patients’ privacy in medical research are rigorous. During the resubmission process we contacted the Patient Data Protection Office at Akershus University Hospital to investigate if an anonymized part of the underlying data could be published with the manuscript, but this request was rejected. However, the data protection office will allow written applications for access to the underlying data if the reader want to reproduce our research findings. We have therefore added a section in the manuscripts informing the reader about this possibility and to whom they can send the data request:

Added to the manuscript at page 20 line 400 to 404:

*“***Availability of underlying data**

Underlying data of this study can be made available for readers who seek to reproduce this study’s results. A written application for data access must be sent to Patient Data Protection Office at Akershus University Hospital. Email address: [Fellesmail.Personvernombud@ahus.no](mailto:Fellesmail.Personvernombud@ahus.no?body=Ikke%20skriv%20sensitiv%20informasjon%20eller%20helseopplysninger%20i%20e-post.)»

We hope this information is sufficient for PLOS ONE to make an exemption from our policies regarding data accessibility.

**5. We are unable to open your Supporting Information file [Letter of confirmation REK]. Please kindly revise as necessary and re-upload.**

Response: We are sorry for the inconvenience. The re-uploaded file of “Letter of confirmation REK” is now in a standard pdf-format and should be readable.

**6. Please include captions for your Supporting Information files at the end of your manuscript, and update any in-text citations to match accordingly. Please see our Supporting Information guidelines for more information: http://journals.plos.org/plosone/s/supporting-information.**

Response: The captions for Supporting Information can now be found at the end of the manuscript after references in accordance with PLOS ONE’s guidelines:

Added to the manuscript at page 23-24 line 534 to 541:

*“* *Supporting information*

*S1 Table. Definitions of extracted variables and diagnostic accuracy estimates.*

*S2 Table. Registered diagnoses of non-deep vein thrombosis patients. List and frequency of all international classification of diseases version 10 (ICD-10) registered in Akershus university hospital medical record system of patients in whom deep vein thrombosis was excluded.*

*S3 Table. Contingency table of D-dimer. Contingency table displaying diagnostic accuracy estimates of D-dimer using the cut-off value of ≥ 0.50 mg/L.”*

**7. Please review your reference list to ensure that it is complete and correct. If you have cited papers that have been retracted, please include the rationale for doing so in the manuscript text, or remove these references and replace them with relevant current references. Any changes to the reference list should be mentioned in the rebuttal letter that accompanies your revised manuscript. If you need to cite a retracted article, indicate the article’s retracted status in the References list and also include a citation and full reference for the retraction notice.**

Response: Thank you for this reminder; we have checked the appearance of the references. No references have been added or removed.

Responses to comments from reviewer 1:

Reviewer #1: Review Comments on the Manuscript: "Complement activation assessed by C3bc and C5b-9 terminal complex as diagnostic biomarkers for deep vein thrombosis"
This manuscript investigates the potential of C3bc and C5b-9 terminal complex (TCC) as diagnostic biomarkers for deep vein thrombosis (DVT) by examining 366 patients. The study demonstrates that the levels of complement activation products C3bc and C5b-9 TCC are elevated in acute DVT. However, their diagnostic performance for DVT, either alone or in combination with D-dimer, is found to be relatively low. The study design is rigorous, but several issues need to be addressed to improve the manuscript:

**1. Confusing Background Section: The background section provides a rather disorganized description of the current diagnostic methods for DVT. It is recommended to optimize and summarize the existing diagnostic approaches and their limitations more clearly. This would help readers better understand the significance of the current study.**

Response: Thank you for this recommendation. We have rewritten the second and third section of the manuscript to give a more organized and comprehensive description of the current diagnostic algorithm of DVT and its limitations.

Rewritten parts of the introduction of manuscript at page 4 and 5 line 60 to 90:

*“Current diagnostic guidelines recommend using a multi-step diagnostic algorithm consisting of clinical pre-test probability assessment (PTP), the D-dimer blood test and radiological procedures, most often compression ultrasound (CUS), to ensure sufficient diagnostic precision and cost-effectiveness for patients with suspected DVT [3-5]. Patients are recommended to be categorized into low PTP or high PTP (simplified Wells score) during history taking and clinical examination, of which patients with low PTP should have their D-dimer levels measured. Patients with low PTP and D-dimer levels below cut-off value can safely be ruled out from further testing and treatment because of D-dimer’s excellent negative predictive value of >95% for DVT in this patient group [3-5]. Although a single blood test is quick and easy to perform, D-dimer's low positive predictive value of under 50% is not sufficient to confirm the DVT diagnosis. CUS has an excellent positive predictive value of over 95% in patients with low PTP and positive D-dimer and for patients with high PTP, and is therefore recommended as the final confirmatory test for DVT suspicion [3-5]. Although CUS does not entail contrast and radiation exposure such as CT venography, it is still time consuming and vulnerable for unavailable equipment and expertise which can lead to diagnostic delay [3, 4]. Therefore, selecting patients in need of CUS using PTP assessments and D-dimer testing is pivotal to ensure the diagnostic algorithm’s cost-effectiveness. When the algorithm for DVT is followed rigorously, it can reduce the number of required CUS procedures by up to 30-40% of all patients with suspected DVT [3, 6, 7].*

*Increasing evidence suggests low compliance to the abovementioned algorithm leading to unnecessary use of CUS due to bypassing of the PTP assessment and D-dimer based selection strategy. Excess use of CUS could lower the test’s positive predictive value of DVT and increase health expenditures, threatening the algorithms diagnostic precision and cost-effectiveness [3-5]. In a recent systematic review, Kraaijpoel et al. reported that only 6-25% of the CUS performed confirmed a DVT [8], indicating low adherence to the diagnostic algorithm. Low adherence to the diagnostic algorithm is supported by data from the GARFIELD-VTE registry, including 8189 DVT-patients from 28 countries, where use of PTP and D-dimer in the diagnostic work-up was reported in only 4.6% and 25.7% of the patients, receptively [9]. Regardless of the underlying reasons for the high rates of performed CUS, it emphasizes potential room for improvement of the current diagnostic strategy for patients with suspected DVT.”*

**2. Missing P-values in Table 1: In Table 1, which presents the demographic and clinical characteristics of included patients, there is a lack of P-values comparing the DVT group and the Non-DVT group. It is essential to add a column for P-values to indicate whether there are statistically significant differences between the two groups for each parameter.**

**3. P-value Annotation in Figure 2: For the results of plasma levels of C3bc, TCC, CRP, and D-dimer in acute DVT presented in Figure 2, it is important to annotate the P-values. For example, use "ns" for non-significant differences, "*" for p<0.05, or directly label the exact P-values.**

Response to comment 2 and 3: In accordance with your suggestions, we have added p-values to table 1 (page 12 and 13) and added p-values to TCC, CRP and D-dimer in Figure 2. In addition, we have added which type of significance test we have added and level of significance in the statistical analyses section.

We added this text to the last section of page 10 line 209 to 214:

*“Whitney U-test was also applied to compare differences in plasma levels of the biomarkers between the two groups in addition to the continuous demographic baseline variables of the study population. The chi-square test was performed on categorical clinical and demographic variables. The significance level was a p-value ≤ 0.05.”*

**4. Limitation of Single-Center Study: The study is conducted in a single center, which inherently limits its generalizability. This limitation should be discussed in the manuscript.**

**5) Additional Limitations in the Discussion Section: The discussion section should include other limitations of the study. The authors should critically evaluate potential biases, sample size issues, or other factors that might have influenced the results.**

Response to comment 4 and 5: Thank you for these remarks, we acknowledge the importance of communicating the limitations of the study comprehensively and clearly. Our intent with the second last section of the discussion was to point out both strengths and weaknesses. However, reading through it again, it is apparent that the description of the study’s weaknesses has been too vaguely described.

We have therefore rewritten parts of the discussion section to highlight weaknesses of our study design. Page 18 to 19 line 354 to 381:

*“The main strength of our study was the prospective consecutive sampling strategy including patients with suspected lower limb DVT to reduce the risk of selection bias. It is pivotal to avoid convenience sampling from two different source populations (i.e., two-gate case-control sampling) to preserve the spectrum of disease severity of DVT as well as the spectrum of alternative diagnoses to DVT [34]. If these spectrums are compromised, the risk of falsely increased sensitivity and specificity is high [34, 36]. The distribution of patients with proximal- and distal DVT, DVT and PE, isolated DVTs and differential/alternative diagnoses of non-DVT patients were comparable to the reported distributions in routine clinical practice [37-39], indicating that disease spectrums were most likely preserved in our study. Still, the prevalence of DVT was somewhat higher than observed in clinical practice, indicating a chance of selection bias where the study cohort of non-DVT-patients are at risk of not being clinically representable. Such selection bias would most likely lead to a falsely high specificity and positive predictive value [40], thus, not impacting the overall negative results of our study. Comparison with only one reference standard (whole leg CUS), and that the personnel assessing the index tests were unaware of the DVT status and vice versa, reduced the risk of information biases such as differential or partial verification bias [36]. Still, as whole leg CUS can be difficult to interpret, especially for distal DVTs, the study is at risk of information bias, imperfect gold standard bias, which could falsely increase the index tests’ sensitivity and negative predictive values [36]. However, only four non-DVT patients had a confirmed VTE within three months of inclusion, indicating a low risk of this bias on our main results. An alternative reference standard could have been CT venography, which has comparable diagnostic accuracy to CUS specificity and is less prone to patient-related factors (such as obesity) and examiner-related factors (such as less experience examiner) [41]. CT venography’s are recommended in clinical guidelines only for a few very selected patients with DVT suspicion as it is more time-consuming than CUS and exposes patients to radiation and contrast medium-induced morbidity [3]. As no other study has investigated the diagnostic utility of complement activation products in the setting of DVT suspicion, we aimed to discover the discriminatory abilities of C3bc and TCC in an unselected group of patients with suspected DVT. Hence, we refrained from investigating the performance of the biomarkers within framework of the recommended diagnostic algorithm of DVT. This is a core limitation of the study; the diagnostic accuracy of the investigated biomarkers cannot be interpreted directly into context of current diagnostic guidelines of DVT suspicion. Furthermore, the generalizability of the study results is limited. First, including only first-time lower-limb DVT reduces the transferability of the results to patients with recurrent DVT and patients with DVT at atypical sites. Second, this study is a single-center study which limits the sample size and exclude differences between clinical practices in various hospitals and countries.”*

**6) Further Discussion on Complement Activation Products: Based on the findings of this study and relevant literature, the authors should provide a more in-depth discussion on why the plasma levels of C3bc and C5b-C9 TCC were elevated in acute DVT but exhibited poor diagnostic performance for DVT, either alone or in combination with D-dimer. This section should explore potential biological reasons and the implications for future research.**

Response: We agree with reviewer 1 that this is an essential part of a solid manuscript. However, we do not fully agree that exploration of potential biological reasons and the implication for future research is not comprehensively enough discussed. We have added a sentence to the second paragraph of the discussion section, going more in-depth of the clinical application of our results, page 19 line 323 to 324.

*“The low AUCs of C3bc and TCC also indicate that the biomarkers are not suitable for a “rule in diagnostic algorithm” to further testing and/or treatment”*

Regarding exploration of potential biological pathways, given that this study is a clinical study, we believe that parts of the second and the entire third paragraph of the discussion give a precise discussion of the core biological pathways of inflammation and coagulation in which C3bc, TCC, CRP and D-dimer are involved providing the reader with sufficient biological fundament to understand the reasoning and impact of our study results. We have highlighted in the second and third paragraph of the discussion that C3bc and TCC are more upstream or ubiquitous in inflammatory pathways, and thus, increased in DVT and in other diseases causing lower limb swelling and/or pain, thereby contributing to their overall low discriminatory power.

Responses to comments from reviewer 2:

**1. Why patients' life-expectancy less than 2 years was one of exclusion criteria - why was this time chosen?**

Response: Thank you for this comment. This study is a part of a larger VTE-biomarkers project including investigation of other biomarkers not related to the complement system. The exclusion criteria of “life expectancy less than 2 years” was to ensure that most of our inclusion patients would be able to complete the 6- and 12-month follow-up appointment for clinical investigation of post-thrombotic syndrome and additional bloodwork.

**2. It is not cleared if missed VTE was searched for in all patients after inclusion in the study.**

Response: Thank you for this comment. It highlights the confusion phrasing of this aspect in the manuscript. We did a 3-month follow-up on only our non-DVT patients to investigate if the physician in the emergency department had overlooked a DVT upon inclusion. Patients with overlooked DVTs will often revisit the same emergency department as their symptoms worsen. Revisits to the hospital would be documented in their medical records and be available for data registration. The purpose of this follow-up was to investigate the rate of misclassification of non-DVT patients as it is known that compression ultrasound may have some limitations regarding its sensitivity and negative predictive value for DVT depending on patient-related factors and the examiners’ experience. We chose a 3-month period because it is a conventional follow-up period in studies of VTE. As compression ultrasound has excellent specificity and positive predictive value, we did not apply a 3-month follow-up period on our DVT-patients as the chances of misclassification of these patients are low.

We changed the first section appearing on page 9 line 180 to 183:

*“Since the sensitivity of CUS may vary depending on patient-related factors and the examinators’ experience, we investigated the proportion of potential CUS misclassification among non-DVT patient by searching in their hospital medical records for missed VTE (DVT and PE) diagnoses within three months after study inclusion date.”*
